# Establishing a clinical service to prevent psychosis: What, how and when? Systematic review

**DOI:** 10.1038/s41398-020-01165-x

**Published:** 2021-01-13

**Authors:** Gonzalo Salazar de Pablo, Andrés Estradé, Marcello Cutroni, Olivier Andlauer, Paolo Fusar-Poli

**Affiliations:** 1 Early Psychosis: Interventions and Clinical-detection (EPIC) Lab, Department of Psychosis Studies, Institute of Psychiatry, Psychology & Neuroscience, King’s College London, London, UK; 2 Institute of Psychiatry and Mental Health, Department of Child and Adolescent Psychiatry, Hospital General Universitario Gregorio Marañón School of Medicine, Universidad Complutense, Instituto de Investigación Sanitaria Gregorio Marañón (IiSGM), CIBERSAM, Madrid, Spain; 3 Department of Clinical and Health Psychology, Catholic University, Montevideo, Uruguay; 4 Department of Brain and Behavioral Sciences, University of Pavia, Pavia, Italy; 5 Heads UP Service, East London NHS Foundation Trust, London, UK; 6 Centre for Psychiatry, Wolfson Institute of Preventive Medicine, Barts and the London School of Medicine and Dentistry, Queen Mary University of London, London, UK; 7 National Institute for Health Research, Maudsley Biomedical Research Centre, South London and Maudsley NHS Foundation Trust, London, UK; 8 OASIS Service, South London and Maudsley NHS Foundation Trust, London, UK

**Keywords:** Schizophrenia, Scientific community

## Abstract

The first rate-limiting step to successfully translate prevention of psychosis in to clinical practice is to establish specialised Clinical High Risk for Psychosis (CHR-P) services. This study systematises the knowledge regarding CHR-P services and provides guidelines for translational implementation. We conducted a PRISMA/MOOSE-compliant (PROSPERO-CRD42020163640) systematic review of Web of Science to identify studies until 4/05/2020 reporting on CHR-P service configuration, outreach strategy and referrals, service user characteristics, interventions, and outcomes. Fifty-six studies (1998–2020) were included, encompassing 51 distinct CHR-P services across 15 countries and a catchment area of 17,252,666 people. Most services (80.4%) consisted of integrated multidisciplinary teams taking care of CHR-P and other patients. Outreach encompassed active (up to 97.6%) or passive (up to 63.4%) approaches: referrals came mostly (90%) from healthcare agencies. CHR-P individuals were more frequently males (57.2%). Most (70.6%) services accepted individuals aged 12–35 years, typically assessed with the CAARMS/SIPS (83.7%). Baseline comorbid mental conditions were reported in two-third (69.5%) of cases, and unemployment in one third (36.6%). Most services provided up to 2-years (72.4%), of clinical monitoring (100%), psychoeducation (81.1%), psychosocial support (73%), family interventions (73%), individual (67.6%) and group (18.9%) psychotherapy, physical health interventions (37.8%), antipsychotics (87.1%), antidepressants (74.2%), anxiolytics (51.6%), and mood stabilisers (38.7%). Outcomes were more frequently ascertained clinically (93.0%) and included: persistence of symptoms/comorbidities (67.4%), transition to psychosis (53.5%), and functional status (48.8%). We provide ten practical recommendations for implementation of CHR-P services. Health service knowledge summarised by the current study will facilitate translational efforts for implementation of CHR-P services worldwide.

## Introduction

The clinical high risk for psychosis (CHR-P) paradigm^1^ represents one of the most established preventive approaches in clinical psychiatry^2^. It originated in Australia around 25 years ago^3^ and since then, it has progressively gained importance^4^. CHR-P individuals are young and accumulate risk factors for the disorders^5–7^, that lead to functional impairments^8^ and attenuated psychotic symptoms^9^. Because of these features, these individuals seek help^10^ at specialised CHR-P mental health services. The detection^11^, prognostic assessment^12^ and preventive treatment^13–16^ in CHR-P individuals^15^ have the potential to maximize the benefits of early interventions in psychosis^17,18^. A recent evidence-based summary by the European College of Neuropsychopharmacology Network for the Prevention of Mental Disorders and Mental Health Promotion^19^ indicated that the first rate-limiting step to prevent psychosis is to establish specialised CHR-P services^20^. Accordingly, several CHR-P services have been implemented worldwide, as recently mapped by the International Early Psychosis Association (IEPA^21^: https://iepa.org.au/list-a-service).

Despite these progresses, health service research in this field has been fragmented to the point that the characteristics (“what”) of a CHR-P service per se are poorly defined. As CHR-P services expand globally^21^, it becomes essential to synthetize the core CHR-P health service features that have been implemented in real-world scenarios. While a CHR-P clinic can be broadly defined as a “multidisciplinary community mental health service that provides treatment and support to people at high risk of developing psychosis” (page 16 from NHS England^22^), this definition remains elusive. Similarly, there is no clear guidance on “how” to integrate different service components. The three main models for delivering CHR-P services include the “stand-alone”, “hub and spoke”, and “integrated” models^22^. While the standalone model works independently from other more generic community mental health teams, in the “hub and spoke” model, dedicated team workers (“spokes”) are based within more generic community teams to route patients needing more intensive services to the central “hub”^23^. In an integrated model, the CHR-P service is completely integrated into the community mental health care. In addition, these models can be combined within broad mental health services enhancing transitional primary care platforms across adolescents and young adults^24^. The additional limitation of knowledge is that the timing (“when”) for preventive approaches, which is reflected by CHR-P entry age criteria is uncertain. While this has been typically set for young people aged 8–40^4^ years, more recent lifespan-inclusive approaches for those under the age of 25 (0–25 years)^25^ models have been piloted.

While previous systematic reviews have addressed these issues for services taking care of patients with a first episode of psychosis^26,27^, CHR-P research has remained mostly “academic” and did not systematically address real-world service characteristics such as: service configuration, outreach strategy and referrals, service user characteristics, interventions, and outcomes. The current systematic review summarizes, for the first time, evidence on these domains to inform the real-world implementation (i.e., what, how, and when) of CHR-P clinical services worldwide.

## Methods

This study (study protocol: PROSPERO CRD42020163640) was conducted in accordance with PRISMA^28^ (eTable 1) checklist.

### Search strategy and selection criteria

A multistep systematic literature search strategy was used to identify relevant articles by two independent researchers (GSP, AE). First, the Web of Science database (Clarivate Analytics) was searched, incorporating the Web of Science Core Collection, BIOSIS Citation Index, KCI-Korean Journal Database, MEDLINE, Russian Science Citation Index, and SciELO Citation Index as well as Cochrane Central Register of Reviews, and Ovid/PsychINFO databases, as well as the OpenGrey database (for grey literature) from inception until 4th May 2020, with no restrictions on language. The following search terms were applied: (“risk” OR “prodrom*” OR “ultra-high risk” OR “clinical high risk” OR “attenuat*” OR “high risk” OR “genetic high risk” OR “risk syndrome” OR “at risk mental state” OR “at-risk mental state” OR “ARMS” OR “risk of progression” OR “schizophrenia” OR “schizoaffective disorder” OR “schizophreniform disorder”) AND (“psychosis”) AND (“prevention” OR “intervention” OR “early intervention” OR “referral” OR “assessment” OR “service” OR “clinical service” OR “psychiatric service” OR “implementation” OR “care pathways”). The references of the articles identified in previous reviews and relevant commentaries and the references from the included studies were manually searched to identify additional relevant records. Abstracts were screened, and potential full texts were assessed against inclusion and exclusion criteria.

The inclusion criteria were a) being an original study published in international databases or in the grey literature, b) describing clinical services for individuals in a CHR-P state as defined according to established instruments: Comprehensive Assessment of At-Risk Mental States (CAARMS^3^), Structured Interview for Psychosis-risk Syndromes (SIPS^29,30^), Bonn Scale for the Assessment of Basic Symptoms (BSABS^31^), Basel Screening Instrument for Psychosis (BSIP^32^), Schizophrenia Proneness Instrument^33^ - Adult (SPI-A) and Child and Youth (SPI-CY) version -, Positive and Negative Syndrome Scale (PANSS^34^), Scale for the Assessment of Negative Symptoms (SANS^35^), Brief Psychiatric Rating Scale (BPRS^36^) and Early Recognition Inventory (ERIraos^37^), c) providing information on any of the following: service configuration, outreach strategy and referrals, service user characteristics, interventions and outcomes, d) providing relevant information without any restrictions on language, sex, age, or ethnicity. The exclusion criteria were a) non-original studies such as abstracts, conference proceedings, study protocols, reviews, guidelines, b) studies with a primary research focus (e.g., research networks) and lacking description of CHR-P clinical services, c) studies describing clinical services for conditions other than the CHR-P or services without a CHR-P component, d) national or regional survey studies with aggregate data and lacking a service-specific description.

### Descriptive measures and data extraction

Independent researchers (GSP, AE, MC) extracted data from the included studies; discrepancies were resolved through consensus, consulting a senior researcher (PFP). The variables included were those necessary to describe “what, how and when” to implement CHR-P services. These variables were grouped according to health service domains previously established (beyond general data such as first author, year of publication, name of the CHR-P service, country)^38^: (i) service configuration: continent, service set-up date, population in the catchment area, type of service, professionals involved, (ii) outreach strategy and referrals: outreach activities—measured using an adapted version of the Longitudinal Youth-At-Risk Study (LYRIKS) study^39^ classification—, referral sources (iii) service user characteristics: sociodemographic characteristics, CHR-P assessment, CHR-P subgroups (defined as in previous studies)^40^, minimum and maximum age inclusion criteria and service use age range, comorbidities and employment (iv) interventions: type of intervention (non-pharmacological vs. psychopharmacological) and duration of service provision and (v) outcomes: type of outcomes monitored and outcome instruments. Furthermore, we reported quality assessment (see below).

### Data analysis

#### Systematic review

All the studies were systematically summarized in tables reporting on various health service domains: service configuration, outreach strategy, and referrals (Table 1), service user characteristics (Table 2), interventions and outcomes (Table 3). We complement this with descriptive analysis of common operational and clinical challenges. An online tool (https://www.maptive.com) was used to create a graphical representation of the geographical distribution of the CHR-P services included in the review.

**Table 1:** Service configuration (above); outreach strategy and referrals (below).

	Number of services (%)
Service configuration	
Continent	51
Europe	30 (58.8)
North America	13 (25.5)
Australia	4 (7.8)
Asia	3 (5.9)
South America	1 (2.0)
Service set-up date	50
1991–1999	6 (12.0)
2000–2009	31 (62.0)
2010–2019	13 (26.0)
Population in the catchment area	35
Combined total population	17,252,666
Average total pop. per service	492,933
Type of service	51
Integrated CHR-P service	41 (80.4)
Standalone CHR-P service	10 (19.6)
Hub and spoke CHR-P service	0
Professionals involved (not mutually exclusive)	30
Psychiatrist^a^	30 (100)
Clinical psychologist or counsellor^b^	23 (76.7)
Case manager/care coordinator^c^	15 (50.0)
Nurse^d^	15 (50.0)
Occupational therapist/social worker/educator	12 (40.0)
Research personnel^e^	3 (10.0)
Neuropsychologist	2 (6.7)
General practitioner	1 (3.3)
Exercise physiologist	1 (3.3)
Outreach strategy and referrals	
Outreach activities (not mutually exclusive)	41
Active approaches	
Workshops	
General workshops	40 (97.6)
Targeting healthcare professionals	35 (85.4)
Targeting education professionals^f^	20 (48.8)
Targeting community organisations	14 (34.1)
Service promotion to NGOs and community services^g^	14 (34.1)
Service promotion to social and governmental services^h^	8 (19.5)
Service promotion to family members	2 (4.9)
General public awareness campaigns	15 (36.6)
Passive approaches	
Dedicated online site	23 (63.4)
Print and other media^i^	22 (53.7)
Referral sources (not mutually exclusive)	40
Outpatient or community mental health services^j^	36 (90.0)
General healthcare^k^	30 (75.0)
Education organisations or services^l^	26 (65.0)
Self	24 (60.0)
Family, relatives or friends	24 (60.0)
Inpatient mental health services^m^	17 (42.5)
A&E departments	9 (22.5)
Social services & welfare	7 (17.5)
Government organisations^n^	6 (15.0)
Community organisations^o^	5 (12.5)
Early Intervention for Psychosis services	5 (12.5)

For footnotes see the supplementary section (eResults 1).

*A&E* Accident and emergency Departments, *NGO* non-governmental organization.

**Table 2:** Service user characteristics.

	Number of services (%)
Sociodemographic characteristics	
Sample size	33
<50	12 (39.4)
50–100	6 (18.2)
>100	15 (45.5)
Sex	43 (CHR-P individuals)
Male (frequency %)	57.2
CHR-P Assessment (not mutually exclusive)	37
CAARMS	18 (48.6)
SIPS	13 (35.1)
ERIraos-CL	6 (16.2)
BSIP	2 (5.4)
BSABS/SPI-A/SPI-CY	1 (2.7)
Min. age inclusion criteria	48
Between 8 and 6 years	2 (4.2)
12 years	12 (25)
Between 13 and 15 years	15 (31.3)
Between 16 and 17 years	13 (27.1)
18 years or older	6 (12.5)
Max. age inclusion criteria	49
18 years	4 (8.2)
Between 24 and 29 years	14 (28.6)
Between 30 and 35 years	24 (49.0)
Between 40 and 56 years	4 (8.2)
65 years or older	3 (6.1)
Service users age range	51
Children and adolescents only (<18)	2 (3.9)
Adolescents only (12–18)	2 (3.9)
Adolescents and young adults (12–35)	36 (70.6)
Children, adolescents and adults (8–40)	1 (2.0)
Adolescents and adults (≥12)^a^	5 (9.8)
Young adults (18–35)	4 (7.8)
Adults only (≥18)	1 (2.0)
	CHR-P individuals (%)
Diagnostic subgroups (not mutually exclusive)	17^b^
APS	82.6
BLIPS	10.7
GRD	8.5
Comorbidity (not mutually exclusive)^c^	31^b^
Any DSM/ICD comorbid disorder	69.5
Depressive disorders	42.3
Bipolar disorder	15.5
Persistent depressive disorder	6.7
History of suicide attempts	10.5
Anxiety disorders	24.1
Social phobia	5.9
Obsessive compulsive disorder	5.1
Adjustment disorder	11.6
Any personality disorder	15.5
Schizotypal personality disorder	11.0
Substance use disorders	12.4
Employment	13^b^
Unemployment rates	36.6

^a^Includes two services that enrolled “adolescents and adults” without further specification.

^b^Number of services providing data for the service user characteristics as % CHR-P individuals.

^c^Diagnosis according to DSM or ICD criteria stablished using either structured interviews or clinical interviews.

*APS* attenuated psychosis symptoms, *BLIPS* brief limited intermittent psychotic symptoms, *BSABS* Bonn scale for the assessment of basic symptoms, *BSIP* Basel screening instrument for psychosis, *CAARMS* comprehensive assessment of at-risk mental states, *ERIraos-CL* early recognition inventory retrospective assessment of symptoms checklist, *GRD* genetic risk and deterioration, *SIPS* structured interview for psychosis-risk syndromes, *SPI-A* schizophrenia proneness instrument (adults version), *SPI-CY* schizophrenia proneness instrument (child and youth version).

**Table 3:** Interventions (above) and outcomes (below).

	Number of services (%)
Interventions	
Non-pharmacological interventions (not mutually exclusive)	37
Clinical monitoring	37 (100.0)
Psychoeducation	30 (81.1%)
Case management and psychosocial support^a^	27 (73.0)
Family intervention^b^	27 (73.0)
CBT-based individual intervention^c^	25 (67.6)
Other individual psychotherapeutic intervention^d^	24 (64.9)
Physical health interventions^e^	14 (37.8)
Group social or therapeutic interventions^f^	7 (18.9)
Pharmacological interventions (not mutually exclusive)	31
Antipsychotic medication	27 (87.1)
Antidepressants	23 (74.2)
Anxiolytics	16 (51.6)
Mood stabilizers	12 (38.7)
Omega-3 fatty acids	3 (9.7)
Duration of service provision	29
6 months	1 (3.4)
12 months	7 (24.1)
24 months	13 (44.8)
36 months	3 (10.3)
60 months or more	5 (17.2)
Outcomes	
Type of outcomes (not mutually exclusive)	43
Persistence of symptoms/comorbidities	29 (67.4)
Transition to psychosis	23 (53.5)
Functional status	21 (48.8)
Remission	18 (41.9)
Physical health outcomes	13 (30.2)
Service users’ satisfaction	11 (25.6)
Hospitalisation	8 (18.6)
Mortality	6 (13.9)
Outcome instruments (not mutually exclusive)	43
Clinical interviews	40 (93.0)
Psychometric instruments	16 (37.2)
CAARMS	10 (23.2)
SIPS	5 (11.6)
Electronic health records	7 (16.3)

For footnotes see the supplementary section (eResults 2).

### Quality assessment

We adapted the mixed Methods Appraisal Tool (MMAT)^41,42^ questions for non-randomized clinical studies due to the heterogeneity expected in the included studies to assess the quality of the included studies (eMethods 1), considering the content and characteristics of the studies according to our inclusion criteria.

## Results

### Database

**Fig. 1:**
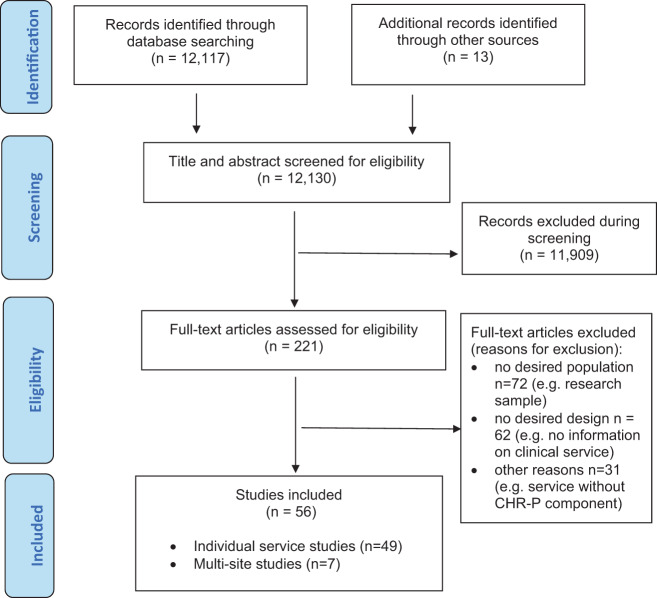
PRISMA Flowchart. Preferred reporting items for systematic reviews and meta-analyses (PRISMA) flowchart outlining study selection process.

**Fig. 2:**
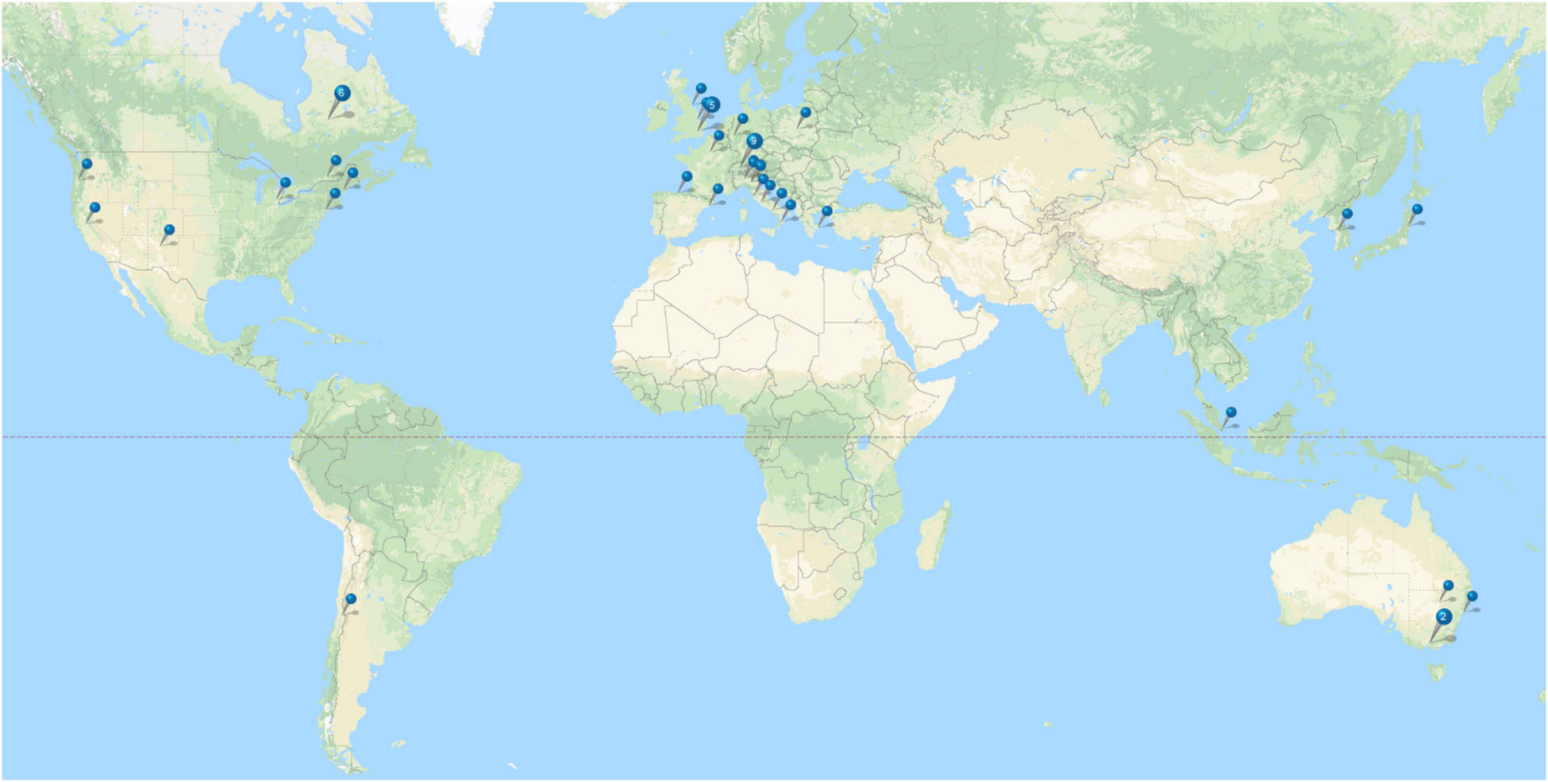
CHR-P services map. Geographical distribution of CHR-P services included in the review.

The literature search yielded 12,130 citations, which were screened for eligibility. Two hundred and twenty-one full-text articles were evaluated for eligibility, and 165 were excluded. In total, 49 studies reporting information on individual CHR-P services (eTable 2), and seven multisite studies (eTable 3) were selected (PRISMA, Fig. 1). All CHR-P services (100%) used validated assessment instruments and no studies were excluded for this reason. The final pool of 56 included studies were published between the years 1998 and 2020. The total sample encompassed 51 distinct CHR-P clinical services, from 41 different regions, across 15 countries (Fig. 2).

Most multisite studies reported on collaborative networks of clinical CHR-P services, including the Pan-London Network for Psychosis-Prevention (PNP^38^), the Early Detection and Intervention for the Prevention of Psychosis Program (EDIPPP^43–45^), and the Swiss Early Psychosis Project (SWEPP^46^). Two additional multisite studies report on five centres operating under the Italian Departments^47^ and six CHR-P services in Canada^48^.

### Service configuration

The CHR-P services were located mostly in Europe (58.8%), followed by North America (25.5%), Australia (7.8%), Asia (5.9%), and South America (2.0%; Fig. 2 and Table 1). The first program to be implemented was the Personal Assessment and Crisis Evaluation (PACE) clinic in 1994, in Melbourne^49^, and the most recent one the City & Hackney At-Risk Mental State Service (HEADS UP) in 2015, in London^38^. 62.0% CHR-P services were set up from 2000 to 2009 (Table 1). Population in the catchment area covered a total of 17,252,666 people, with an average of 492,933 people (SD: 396,997, Table 1). Most services (80.4%) consisted of teams integrated into the community mental health care. Standalone CHR-P services were less frequent (19.6%) and there were no hub and spoke services. CHR-P clinical services involve a wide range of professionals, the most frequent ones being psychiatrists (100%), who were involved in all the services. Other professionals include clinical psychologists or counsellors (76.7%), case managers/care coordinators (50%), and nurses (50%).

### Outreach strategy and referrals

Outreach activities and audiences were highly variable (Table 1). Within active strategies, workshops for referral sources were the most frequent (97.6%), often targeting healthcare professionals (85.4%), educational professionals (48.8%), or community organisations (34.1%). Services also approached NGOs and community services (34.1%), and less frequently social and governmental services (19.5%) and family members of mental health patients (4.9%). About one-third of CHR-P services (36.6%) implemented general public awareness campaigns including TV or radio appearances^50,51^, theatre adverts, high school art contests, and sponsors for minor league sports teams^45^. More than half of services (63.4%) implemented either a dedicated online site for service promotion, or have elaborated printed and other media materials (53.7%), such as information brochures and leaflets^50–52^, posters^53^, articles in professional journals and local newspapers^51,54^, presentations in scientific conferences^46^, newsletters^51^, and promotional videos^43,55^. Most CHR-P services received young people with a putative risk of psychosis from health-related organizations, including both outpatient or community mental health services (90.0%) and general healthcare services (75.0%). Education organisations are also frequent referral sources (65.0%), followed by self (60.0%), family or relatives (60.0%), inpatient mental health services (42.5%), and accident and emergency departments (22.5%). Other referral sources were reported in less than 20% of CHR-P services.

### Service user characteristics

The total sample size of service users was of 5637 CHR-P individuals, ranging from 4^56^ to 467^57^ individuals: most of them were males (% of CHR-P females = 42.8, see Table 2).

CHR-P status was most frequently assessed using the Comprehensive Assessment of At-Risk Mental States (CAARMS) (48.6%), followed by the Structured Interview for Psychosis-risk Syndromes (SIPS) (35.1%) and the Early Recognition Inventory retrospective assessment of symptoms checklist (ERIraos-CL) (16.2%; Table 2). The Basel screening instrument for psychosis (BSIP) and basic symptoms instruments were infrequently used (<6% of services). Most services provided treatment starting in adolescence, from the ages 12 to 17 (83.4%). The most frequent minimum inclusion age range was 13–15 years (31.3%); only two services reported the inclusion of children from the age of 8 and 6^46,48,58^. Most services accepted users until 30–35 years (49.0%) or 24–29 years (28.6%). A few services (6.1%) accepted service-users of 65 years or older^56,59^. The most frequent age range (70.6% of services) was 12 and 35 years.

82.6% of the CHR-P individuals fulfilled APS criteria, 10.7% fulfilled BLIPS criteria and 8.5% fulfilled GRD criteria (not mutually exclusive). Baseline comorbid mental disorders were reported in 69.5% CHR-P individuals. Mood disorders were the most common: depressive disorders (42.3%), bipolar disorders (15.5%), and persistent depressive disorder (6.7%). Anxiety disorders were also frequent (24.1%), including social phobia (5.9%) and obsessive–compulsive disorder (OCD) (5.1%). Adjustment disorder appeared in 11.6% of CHR-P individuals. Comorbid personality disorders were present in 15.5% of CHR-P individuals, particularly schizotypal personality disorder (11.0%). Substance use disorders were present in 12.4% of CHR-P individuals. Past history of suicide attempts was present in 10.5% of CHR-P subjects. Unemployment rate (i.e., neither work nor study) was observed in 36.6% in CHR-P individuals in clinical services.

### Interventions

Across non-pharmacological interventions, clinical monitoring was the most common intervention and was carried out in all the services (100%). Other common interventions were psychoeducation (81.1%), case management and psychosocial support (73.0%) and family interventions (73.0%) (Table 3). Cognitive behavioural therapy-based interventions and any other type of individual psychotherapeutic intervention (encompassing individual motivational interviewing^60^ or sessions^47^, supportive counselling^38,44,47,49,52,61–71^, relaxation training^61^, cognitive remediation^55^, solution focused brief therapy^55^, social skills training^65,70^, substance misuse work^70^, and psychotherapy NOS^72,73^) were provided by about two-thirds of the services (67.6% and 64.9%, respectively). Physical health interventions and group psychotherapy sessions were more infrequent (37.8% and 18.9%, respectively).

Most (87.1%) services employed low-dose antipsychotic (AP) medication, although not as the first-line intervention but only following worsening of symptoms or functioning^52,74,75^. Other interventions employed by CHR-P services included antidepressants (74.2%), anxiolytics (51.6%), mood stabilisers (38.7%). Three services (9.7%) reported the use of omega-3 fatty acids^38,66,71,76,77^. Most services provided care for 24 months (44.8%) or 12 months (24.1%). Three services (10.3%) provided 36 months of clinical follow-up. Extended service provision of 60 months or more were reported in 17.2% of services.

### Outcomes

The outcomes most frequently evaluated in the CHR-P services were persistence of symptoms/comorbidities (67.4%), transition to psychosis (53.5%), functional status (48.8%), and remission (41.9%). Physical health outcomes (30.2%), service users’ satisfaction (25.6%), hospitalisation (18.6%) and mortality (13.9%) were less frequently evaluated.

Outcomes were most commonly evaluated with standard clinical interviews with the service users (93.0%), and more infrequently with psychometric instruments (37.2%). In the latter case, the CAARMS (23.2%) and SIPS (11.6%) were more frequently employed. About 16.3% of CHR-P services evaluated outcomes via electronic health records.

### Quality of the included studies

Study quality scores ranged from 1 to 5. The overall mean quality score for included studies reporting on individual services was 3.8 (moderately high quality) on the MMAT scale, with a SD of 1.3 (eTable 2).

## Discussion

To our knowledge, this is the first systematic review to comprehensively summarize the evidence from real-world implementation of CHR-P clinical services. This review encompasses 56 studies describing a total of 51 services for CHR-P individuals.

Consistent with recent surveys of CHR-P services^21,47,78^, there is great diversity in how clinical services have been implemented in real-world scenarios, across all aspects of service delivery: 1) service configuration, 2) outreach strategy and referrals, 3) service user characteristics, 4) interventions and 5) outcomes. We discuss these points, while also mentioning common challenges. The evidence summarised will then be used to operationalise ten empirical recommendations for overcoming these challenges and facilitating the real-world implementation of CHR-P services (Table 4).

**Table 4:** Ten simple recommendations for real-world implementation of CHR-P service.

Service configuration	
1	Implement a standalone community service (“what”)
2	Train a multidisciplinary team (psychiatrists, clinical psychologists or counsellors, case managers and nurses) (“what”)
Outreach strategy and referrals	
3	Adopt active and passive outreach, primarily targeting healthcare agencies (“how”)
4	Ensure adequate risk enrichment during the recruitment (“how”)
CHR-P service user characteristics	
5	Define CHR-P through established psychometric instruments (not in general population) (“how”)
6	Implement a transitional and transdiagnostic service across adolescents and young adults (“when”)
Interventions	
7	Offer needs-based interventions and psychological interventions (“how”)
8	Titrate the intervention according to the characteristics and risk profile^a^ as well as the values and preferences of the individuals (“how”)
Outcomes	
9	Collect information and target recovery, physical health outcomes, service users’ satisfaction, functioning and quality of life (“how”)
10	Extend clinical monitoring for outcomes for at least three years (“how”)

^a^CHR-P subgroups BLIPS > APS > GRD, severity of attenuated positive and negative symptoms, and level of functioning.

In terms of service configuration, several CHR-P clinical services have been implemented across—at least—15 countries (Fig. 2), covering a catchment area of over 17 M people. Following a period of rapid expansion (2000–2009), new CHR-P services continue to emerge^38,76^. At present, CHR-P services spread across most continents^21^, although they are mostly established in high-income countries. While most CHR-P services are configured as integrated services (80.4%), standalone models of care (19.6%) seem to be associated with high levels of service efficiency^27^. For example, CHR-P standalone services had dropout rates in the range of 12–19.2%^50,77,79–82^ compared to 25.4% in integrated services^52^. One possible explanation is that in integrated models of care, healthcare resources are typically diverted towards more severe service users (e.g., first-episode vs. CHR-P patients)^38^. In line with this notion, the actual caseload of CHR-P individuals was minimal (*n* = 4 out of 239 clients) in some integrated services^56^, and more severe patients had more frequent contacts with these services^73^. Another issue is that standalone services may be physically located outside general psychiatric services, which is preferable to reduce stigmatisation risks^77,81^. Service users and their relatives were generally more satisfied with standalone CHR-P services, particularly with clinical contact being outside traditional mental health settings^67^. Conversely, family disengagement was the most significant barrier (71.4%) in integrated services^60^. Likewise, primary care clinicians favoured standalone models of care because of the superior accessibility of the services^67^. Standalone services are more costly to set up in the first year but deliver highest economic savings in the longer term^69^, mainly associated with the improved outcome of the disorder^68^. These considerations are of relevance given that poor financial support and lack of adequate infrastructures are frequently cited barriers for the establishment of standalone CHR-P services outside mental healthcare^21^. Future health service research is expected to consolidate these speculations, as well as to test the efficiency of innovative models of care. For example, although there were no hub and spoke services, this organization design, which arranges service delivery assets into a network, may be particularly promising in this field and fit well with the youth friendly mental health reform which is undergoing in several countries^83^. Based on this evidence we recommend to preferably implement standalone services (Table 4).

This review also indicates that the CHR-P clinical services are essentially multidisciplinary, reflecting the complexity of the psychopathological assessment and case formulation^84^. Based on the most frequent professionals involved in CHR-P services, we recommend a minimum team encompassing psychiatrists, clinical psychologists, or counsellors, case managers and nurses (Table 4). Because multidisciplinary work requires adequate articulation and training of staff, a core associated recommendation is to ensure adequate training^85^. National surveys have found lack of specialised training in evidence-based interventions to cause dismal across staff^78,86^. Ensuring proper training is particularly challenging for non-academic services, with less resources and limited organizational support^48^.

In terms of outreach strategy, school, mental health, and physical health practitioners were the core targets of community outreach^43^. We have confirmed high heterogeneity across two main strategies: active (up to 97.6%) and passive (up to 63.4%) outreach. The first strategy involved active efforts to organise workshops more frequently targeting healthcare professionals (85.4%), or service promotion activities in the community (up to 34.1% of CHR-P services) and implementing general public awareness campaigns (36.6%). The second strategy involved passive approaches such as a dedicated online site (63.4%), or printed and other media materials (53.7%). This heterogeneity is likely to reflect diverse culturally sensitive approaches across CHR-P services that led to variable pathways to care. In terms of referrals, most CHR-P services received young people with a putative risk of psychosis from health-related organisations such as mental health services (90.0%) and general healthcare services (75.0%). Implementing an outreach to promote referrals of CHR-P individuals is challenging. In the lack of clear guidance, there is high risk of inefficient use of resources (e.g., staff) and inappropriate referrals that eventually do not meet CHR-P criteria. For example, some CHR-P services reported a high number of inappropriate referrals following intense media campaigns, switching to more focused outreach strategies^49,51,61,79,87–90^. At times of financial constraints, the core outreach activities and referral targets summarised in the current study can be used as benchmark to maximise the efficiency of resources when implementing a new CHR-P service. There are also empirical constraints. For example, difficulties in recruiting participants is the most difficult challenge in countries where the CHR-P paradigm is starting to be implemented^76^ and in culturally diverse catchment areas^43^. Even in countries with an established CHR-P network like the UK, increasing numbers of referrals following the implementation of new national policies resulted in more dedicated CHR-P services that were needed to manage the referrals^59^. Finally, the type of outreach and referrals determine the accumulation of established risk factors for psychosis^5,7,91^, thus influencing the level of psychosis risk among individuals recruited for undergoing a CHR-P assessment (also termed as pretest risk enrichment)^92,93^. For example, individuals sampled from inpatient units may have accumulated more risk factors for psychosis and therefore present with a higher level of psychosis compared to those sampled from the community. This level of risk enrichment^93,94^, substantially impacts the clinical utility of CHR-P instruments^12^. Accordingly, intense outreach strategies mainly targeting the general population end up diluting the level of pretest psychosis risk^93^, and therefore impeding a clinically meaningful identification of CHR-P individuals^11,95^. In line with recent psychometric guidances^12,20^, we recommend CHR-P outreach to primarily target healthcare agencies to promote referrals from these sources (Table 4). Community outreach and recruitment from the general public should be considered only if adequate risk enrichment strategies can be implemented (for a detailed review see ref. ^11^). For example, pre-screening approaches can increase pretest risk enrichment among referrals^21^ and was employed by some services^47^.

In terms of service users characteristics, we confirmed that males were relatively more represented than females, in line with the epidemiological gender distribution of psychosis risk^6^. Currently, the vast majority (83.7%) of CHR-P services employ the CAARMS or the SIPS, while basic symptoms instruments failed to enter clinical practice at large scale. This suggests that the harmonisation of these two instruments could deliver a widely used gold standard assessment measure for clinical practice. A rapid response to referrals^62^ and flexibility with time and setting of assessments^67^ have been found to improve engagement with CHR-P services.

Age intake is a core implicit criterion (along with the help-seeking behaviour) defining the CHR-P state^4,10,20^. The most frequently applied age range (70.6% of services) was of 12–35 years, in line with epidemiological research indicating that the peak of risk is between 15 and 35 years^6^. Empirical research confirms that CHR-P psychometric assessment (e.g., the CAARMS) is valid in young people aged 12 years upwards^64^. This finding also confirms the transitional nature of the CHR-P paradigm that cuts across adults and children and adolescent mental health services^25^. Accordingly, most services provided treatment starting in adolescence (between 13 and 15 years). Conversely, only a few services accepted users beyond 40 years^56,59,96^. The requirement of extending the assessment and care of emerging psychosis in the older people, introduced by national guidelines such as the Access and Waiting Time Standards in the UK^22^ is against the evidence that CHR-P instruments are valid up to 40 years^4^. Furthermore, it conflicts with recent mental health reforms that are lowering—as opposed to increasing—the age threshold for preventive approaches to those aged from 0 to 25 years^25^. Based on these findings we recommend that CHR-P services ascertain the at-risk status through the CAARMS or SIPS in both adolescents and young adults (Table 4). This review also indicated that presentation to CHR-P services was associated with frequent comorbid mental health conditions (in particular mood and anxiety disorders^97,98^) in two-thirds (69.5%) of the individuals, coupled with past history of suicide attempts in about one in ten (10.5%) and unemployment in about one third (36.6%) of cases. We further observed regional heterogeneity in clinical presentation: substance misuse was more prevalent in Western services^38,51,54,60,65^, while non-existent in Japan^52^. These findings recommend that CHR-P services should adopt a broader “transdiagnostic” approach”^99–101^, which is cutting across several psychopathological dimensions (Table 4), given that psychosis onset can occur from preceding mood dysregulation^102^ or substance abuse. This recommendation is also relevant for current operationalisations of at-risk syndromes, which require formulating a differential diagnosis between psychosis risk and other psychopathological dimensions such as the SIPS or the DSM-5-APS^2^. Although psychotic experiences are frequent in the general population^103,104^, clinical attenuated psychotic symptoms are infrequent and not normally distributed. Only 0.3% of the general young population meet DSM-5-APS criteria^2,105^.

In terms of interventions, most services (72.4%) provided care for 2 years or less (see outcomes below), with some exceptions^38,48,52,54,55,62,63,72,74,81,87,106^, encompassing clinical monitoring (100%), psychoeducation (81.1%), psychosocial support (73%), family interventions (73%), CBT-based individual interventions (67.6%), group psychotherapy (18.9%), physical health interventions (37.8%), antipsychotics (87.1%), antidepressants (74.2%), anxiolytics (51.6%), and mood stabilisers (38.7%). It appears that CHR-P clinical services currently provide a wide range of psychosocial and biological interventions to meet the clinical needs of CHR-P service users. Clinical monitoring, case management and targeted case management are essential elements of preventive treatment^22^, based on the principles of social psychiatry and the importance of engaging CHR-P individuals with healthcare services^107^. These often included psychoeducation and informing patients about their risk, as done in other preventive approaches in medicine^84^. Despite current guidelines recommending psychological interventions (such as cognitive behavioural therapy) as first-line treatment, about one-third of CHR-P services did not provide them. Evidence to favour psychotherapy over other types of interventions in this population is currently uncertain^13,15,16,20^. Conversely, antipsychotic treatment, which is discouraged by current treatment guidelines, was frequently considered, although typically at low dosages and only when the symptoms were deteriorating. This is consistent with data from global and national surveys of CHR-P services^21,78^ that report frequent use of antipsychotic drugs. The relatively frequent use of anxiolytics, antidepressants, and mood stabilizers—which is not considered by current guidelines—can index the transdiagnostic nature of the CHR-P state with frequent affective and anxiety comorbidities. The variety in provision of treatments likely reflects the high clinical heterogeneity of this population and the lack of clear treatment guidelines stratified on their individual needs. For example, current guidelines are not stratified across CHR-P subgroups. Individuals with brief psychotic episodes may be defined through research-based operationalisations, such as brief and limited intermittent psychotic symptoms (BLIPS) or standard psychiatric classifications including “Acute and Transient Psychotic Disorder” as per ICD-11 or DSM-5 “Brief Psychotic Disorder”. There is diagnostic and prognostic overlap across these definitions of brief psychotic episodes^108,109^. Individuals with brief psychotic episodes have the highest risk of developing psychosis^20^—especially when recurrent or presenting with seriously disorganizing or dangerous features—^108,110^. They also display poor clinical outcomes and do not engage with the recommended cognitive behavioural therapy^40,108^, leaving them with unmet need for care^110^. Stratification across these clinical subgroups has been proposed in recent revisions of the CHR-P paradigm^1,20^ and should be considered in future clinical guidelines^21^. Because the uncertainty of current evidence is high, we align with the recent recommendations of the European College of Neuropsychopharmacology Prevention of Mental Disorders and Mental Health Promotion Network^19^ to still offer needs-based interventions and psychological interventions, titrating the intervention according to the characteristics and risk profile (i.e., transition risk, symptom severity, and functional impairment)^20^ as well as the values and preferences of the CHR-P individuals (Table 4)^20^. For example, it seems important to individualise physical health and lifestyle interventions on the needs presented by each service user^60,76^.

In terms of outcomes, surprisingly, persistence of symptoms/comorbidities (67.4%) was measured more frequently than transition to psychosis (53.5%), functional status (48.8%), and remission (41.9%). This likely reflects the efforts of CHR-P services to treat comorbid conditions, aiming for improving recovery, functioning, and quality of life^20^. At the same time, other outcomes such as physical health were collected in only about a third (30.2%) of CHR-P services^111^. CHR-P individuals accumulate genetic and environmental risk factors^20^, including cardiometabolic risk factors as decreased physical activity^112^ and high rates of substance use^112^, including tobacco^112^, alcohol^112^, and cannabis^113^. Thus, more attention should be paid to recording the physical health of CHR-P individuals in clinical services^114^. Another domain of improvement includes a more frequent monitoring of service users’ satisfaction, which is pivotal to higher engagement and decreased drop-out rates. Furthermore CHR-P services should also more extensively monitor healthcare utilisation (e.g., hospital admissions)^115^ and broad outcomes such as mortality rates to better characterise the overall burden of this condition^116^. Future research is needed to standardise a core outcome set for CHR-P research and therefore facilitate collaborative efforts. These initiative should also indicate the assessment measures to be employed to monitor outcomes. Currently, clinical outcomes in CHR-P services are most commonly evaluated with standard clinical interviews (93.0%), and psychometric instruments are more infrequently used (37.2% of cases, most frequently CAARMS or SIPS to evaluate transition to psychosis)^20^. In the future, monitoring broad health outcomes in CHR-P services could leverage electronic health records that can provide real-world, real-time valuable clinical information^11,117–120^ and that are being increasingly implemented in healthcare providers. As noted above, duration of care including clinical monitoring is currently limited to, most frequently (44.8%), 2 years. However, accumulating evidence has clearly indicated that although the risk of psychosis onset peaks within 2 years^121^, it can increase in the longer term at least until 3–4 years^40,122,123^. In addition, non-transitioning CHR-P individuals can continue to experience functional impairment and symptomatology at 6-years^97^. This confirms that a 2-year service provision is insufficient^21^. As such, we recommend clinical monitoring for outcomes to be implemented for at least 3 years (Table 4). Flexible follow-up after this timepoint can help make more efficient use of clinical resources, while tailoring interventions to users’ needs^124^. For example, the clinical follow-up can be extended if service users are still symptomatic or present socio-occupational difficulties^55,62^. Finally, CHR-P services should be prepared to collect information and target outcomes other than psychosis such as recovery, physical health outcomes, service users’ satisfaction, functioning, and quality of life^20,124^. Harmonisation of core outcome set for CHR-P services is a clinical research priority for the future. Several national and regional networks of CHR-P services started to emerge during the decade of 2010–2019 (e.g., EUGEI, PRONIA, PSYSCAN, NAPLS, PNC, HARMONY, PRONET, and STEP) and may facilitate this enterprise, allowing services to leverage best practices and expertise, increasing lobby capacity and enhancing collaborative efforts^38,44,46^. International clinical research infrastructures have also been developed such as the European College of Neuropsychopharmacology Network for the Prevention of Mental Disorders and Mental Health Promotion (ECNP PMD-MHP)^19^. These initiatives will introduce several innovations in the CHR-P field, encompassing personalised prediction of outcomes and individualised interventions, digital screening for improving detection of psychosis risk and enhancement of transdiagnostic research capability within CHR-P services (e.g., preventive interventions for bipolar risk)^125^.

### Limitations

The main limitation of this study is that health service information was scattered across services, and that there are no established standards to measure the core domains. This limited the capacity to quantitatively compare the different services with meta-analyses. Future harmonisation efforts in terms of CHR-P healthcare research would be extremely valuable. The database was nonetheless large and sufficiently powered to analyse different factors including service configuration, outreach strategy and referrals, CHR-P service user characteristics, interventions, and outcomes. Another limitation is the limited knowledge provided about the long-term outcomes. Furthermore, our results are based on data from the literature that has been published. However, some clinical services may be running but not publishing details about service configuration, outreach strategy and referrals, service user characteristics, interventions, and outcomes. At the same time, we hope that our review will stimulate the establishment of a global network of CHR-P services with shared clinical research infrastructures^21^. Finally, a considerable amount of studies were carried out in relatively small samples, with only 45.5% services^39,50–52,59,62,68,69,73,75,80,82,87–90,106,126–128^ including more than 100 CHR-P individuals.

## Conclusions

Health service knowledge summarised by the current study will facilitate translational efforts for implementation of CHR-P services worldwide.

## Supplementary information

Supplementary material
