# Supplementary material for: Establishing a clinical service to prevent psychosis: What, how and when? Systematic review
Source: Transl Psychiatry. 2021 Jan 13;11:43. doi: 10.1038/s41398-020-01165-x (PMC7807021; doi:10.1038/s41398-020-01165-x)
Supplement: Supplementary file 1 — Supplementary material [file 41398_2020_1165_MOESM1_ESM.docx]

**SUPPLEMENTARY MATERIAL**

**eTable 1:** PRISMA statement and checklist……………………..…………………..…………………..…………………..…………………..…………………..…………..page 2-3

**eTable 2:** Characteristics of the included studies from individual programs...…………………..…………………..…………………..……………………….page 4-13

**eTable 3:** Characteristics collaborative studies…………………..…………………..…………………..…………………..…………………..…………………..………..page 14-16

**eMethods 1:** Mixed Methods Appraisal Tool…………………..…………………..…………………..…………………..…………………..…………………..…………..page 17

**eResults 1:** Details about service configuration, outreach strategy and referrals………..…………………..………………………..……………….……….page 18-19

**eResults 2:** Details about interventions and outcomes..…………..…….…………………………………………………..………………………..…………………….page 20

**This supplementary material has been provided by the authors to give readers additional information about their work.**

**eTable 1: Prisma statement and checklist**

| **Section/topic** | **#** | **Checklist item** | **Reported**  **in page** |
| --- | --- | --- | --- |
| **TITLE** | | |  |
| Title | 1 | Identify the report as a systematic review, meta-analysis, or both. | 1 |
| **ABSTRACT** | | |  |
| Structured summary | 2 | Provide a structured summary including, as applicable: background; objectives; data sources; study eligibility criteria, participants, and interventions; study appraisal and synthesis methods; results; limitations; conclusions and implications of key findings; systematic review registration number. | 2 |
| **INTRODUCTION** | | |  |
| Rationale | 3 | Describe the rationale for the review in the context of what is already known. | 3-4 |
| Objectives | 4 | Provide an explicit statement of questions being addressed with reference to participants, interventions, comparisons, outcomes, and study design (PICOS). | 3-4 |
| **METHODS** | | |  |
| Protocol and registration | 5 | Indicate if a review protocol exists, if and where it can be accessed (e.g., Web address), and, if available, provide registration information including registration number. | 4 |
| Eligibility criteria | 6 | Specify study characteristics (e.g., PICOS, length of follow-up) and report characteristics (e.g., years considered, language, publication status) used as criteria for eligibility, giving rationale. | 4-5 |
| Information sources | 7 | Describe all information sources (e.g., databases with dates of coverage, contact with study authors to identify additional studies) in the search and date last searched. | 4 |
| Search | 8 | Present full electronic search strategy for at least one database, including any limits used, such that it could be repeated. | 4 |
| Study selection | 9 | State the process for selecting studies (i.e., screening, eligibility, included in systematic review, and, if applicable, included in the meta-analysis). | 4-5 |
| Data collection process | 10 | Describe method of data extraction from reports (e.g., piloted forms, independently, in duplicate) and any processes for obtaining and confirming data from investigators. | 5 |
| Data items | 11 | List and define all variables for which data were sought (e.g., PICOS, funding sources) and any assumptions and simplifications made. | 5 |
| Risk of bias in individual studies | 12 | Describe methods used for assessing risk of bias of individual studies (including specification of whether this was done at the study or outcome level), and how this information is to be used in any data synthesis. | 6, e17 |
| Summary measures | 13 | State the principal summary measures | 5-6 |
| Risk of bias across studies | 15 | Specify any assessment of risk of bias (i.e. Newcastle-Ottawa Scale (NOS), that may affect the cumulative evidence. | 6, e17 |
| Additional analyses | 16 | Describe methods of additional analyses (e.g., sensitivity or subgroup analyses, meta-regression), if done, indicating which were pre-specified. | N.a. |
| **RESULTS** |  |  |  |
| Study selection | 17 | Give numbers of studies screened, assessed for eligibility, and included in the review, with reasons for exclusions at each stage, ideally with a flow diagram. | 6, figure 1 |
| Study characteristics | 18 | For each study, present characteristics for which data were extracted (e.g., study size, PICOS, follow-up period) and provide the citations. | e4-16 |
| Risk of bias within studies | 19 | Present data on risk of bias of each study and, if available, any outcome level assessment (see item 12). | e4-13 |
| Results of individual studies | 20 | For all outcomes considered (benefits or harms), present a summary data for each intervention group. | e4-13 |
| Synthesis of results | 21 | Present results of study analyzed | 6-9 |
| Risk of bias across studies | 22 | Present results of any assessment of risk of bias across studies (see Item 15). | 9 |
| Additional analysis | 23 | Give results of additional analyses, if done (e.g., sensitivity or subgroup analyses, meta-regression [see Item 16]). | N.a. |
| **DISCUSSION** |  |  |  |
| Summary of evidence | 24 | Summarize the main findings including the strength of evidence for each main outcome; consider their relevance to key groups (e.g., healthcare providers, users, and policy makers). | 9-15 |
| Limitations | 25 | Discuss limitations at study and outcome level (e.g., risk of bias), and at review-level (e.g., incomplete retrieval of identified research, reporting bias). | 15 |
| Conclusions | 26 | Provide a general interpretation of the results in the context of other evidence, and implications for future research. | 15 |
| **FUNDING** |  |  |  |
| Funding | 27 | Describe sources of funding for the systematic review and other support; role of funders for the systematic review. | 15 |

**eTable 2: Characteristics of the included studies from individual programs**

| **Author, year** | **Name of the CHR-P Service** | **Country** | **CHR-P Users description** | | | | | **MMAT** | **Key findings** |
| --- | --- | --- | --- | --- | --- | --- | --- | --- | --- |
|  |  |  | **Sample size** | **Subgroups**^a^ | **CHR-P assessment tool** | **Sex**  **(% male)** | **Age: mean, SD (range)** |  |  |
| Oppetit et al, 2018^1^ | Evaluation Centre for Young  Adults and Adolescents (C’JAAD) | France | 73 | 89% APS, 11% GRD | CAARMS | 67.1 | 21.1, 3.7 (15-30) | 3 | 37% CHR-P individuals suffered depression, 24% anxiety, 15% personality disorders, 7% OCD and 2% eating disorders. 16% CHR-P had no additional symptoms besides CHR-P. SOFAS score was 48.9 (serious impairment in social, occupational, or school functioning). |
| Spada et al, 2016^2^ | C. Mondino National Neurological Institute | Italy | 22 | 77.3% APS, 4.5 % BLIPS, 18.2% GRD | CAARMS | 52 | 15.6, 1.54 (12-18) | 4 | 1-year transition risk of psychosis in the CHR-P group was 26.7 %. Mean transition time was 111 days. 90% CHR-P suffered at least one axis I or axis II comorbidity: 50% of them major depression and 23% anxiety disorders. |
| Pruessner et al, 2017^3^ | Clinic for Assessment of Youth at Risk (CAYR) | Canada | 177 | 80.8% APS, 5.1% BLIPS, 14.1% GRD | CAARMS | 55.9 | 19.3, 4 (14-35) | 5 | No form was required to be evaluated in the service. An initial response to the request was provided within a maximum of 72 hours. 11.3% individuals transitioned to psychosis; 80% of them in the first year. |
| Qujada et al, 2010^4^ | Early Care Equipment for At-Risk of Psychosis Patients (ECEARP) | Spain | 20 | 60% APS, 40% BLIPS, 20% GRD | SIPS/SOPS, ERIraos, | 60 | 15.8 (12-56) | 3 | Estimated incidence of CHR-P was 2.4 cases per year per 10000 inhabitants. Most frequent symptoms at baseline were depressed mood, poor work and school performance, difficulty to start or maintain social relationships and social withdrawal. |
| Vallina Fernandez 2003^5^ | Unidad Salud Mental Cantabria | Spain | 11 | N.a. | SIPS/SOPS, PANSS | N.a. | (16-30) | 1 | 12% CHR-P individuals developed psychosis after two years. |
| Kollias et al, 2018^6^ | Eginitio University Hospital Early Intervention Service | Greece | 26 | 76.9% APS, 11.5% BLIPS, 11.5% GRD | CAARMS | 53.8 | 25.3, 4.3 (15-40) | 4 | Comorbidity in CHR-P included: 19% OCD, 8% social phobia/panic disorder and 4% personality disorder. 50% received low dose antipsychotics and 23% received antidepressants. 19.2% made a transition to psychosis at 3-years of follow up. Mean delay between the onset of frank psychosis and the initiation of treatment for psychosis (DUP) was 15 days. |
| Leanza 2020^7^ | Fruherke-nnung von Psychosen (FePsy) Basel | Switzerland | 200 | 67.5% APS, 7.5% BLIP, 24% GRD | BSIP | 69 | 25.1± 6.9 | 4 | CHR-P individuals had moderate functional impairment. 77.4% requested to discontinue follow-up assessments due to symptomatic improvement (14.6%), transition to other service or psychotherapist (7.3%), lack of time and interest (9.8%), being annoyed by requests for study participations (19.5%). Transition risk was as 15% after one year, 18% after two years, 22% after three years and 28% after five years. The drop-out and disengagement rate of 36% after five years. |
| Riecher-Rossler et al, 2007^8^ | Fruherke-nnung von Psychosen (FePsy) Basel | Switzerland | 58 | N.a. | BSIP, BPRS, SANS | 58.6 | 26.8, 8.9 (>18) | 5 | 40.8% CHR-P did not consent to take part. Drop-out rate during follow-up was 13.8%. 6-month transition rate was 13%, 12-month transition 23%, 24-month transition 25%- and 60-months transition 32%. |
| Schultze‑  Lutter et al, 2017^9^ | Bern Early Detection and Intervention Center for Mental Crises (FETZ, Bern) | Switzerland | 35 | 94.3% APS, 5.7% BLIPS | SIPS/SOPS | 57.1 | 17.5 (8-40) | 4 | Most frequent non perceptual symptoms were unusual thought contents (39.3%) and persecutory ideas (35.3%)., Hallucinations were more frequent in younger individuals <16 years. |
| Theodoridou 2014^10^ | Zurich Program for Sustainable Development of Mental Health Services (ZInEP) | Switzerland | 221 | N.a. | SIPS/SOPS, SPI, PANSS | N.a. | (13-35) | 4 | Outreach activities included local workshops, articles in professional journals, flyers and websites. |
| Schultze‑  Lutter et al, 2009^11^ | Cologne Early Recognition and Intervention Centre for mental crises (FETZ Cologne) | Germany | 292 | 69.4% APS, 21.1% BLIPS, 10% GRD | BSABS, SPI-A, SIPS/SOPS | 61.6 | 24.8, 6.1 (16-40) | 5 | 46% of the calls received by the service were made by patients, 37.5% by relatives and 13.9% by institutions or professionals. Appointments were requested in 67.2% of the messages received. |
| Rao et al, 2013^12^ | Support for Wellness Achievement Programme (SWAP) | Singapore | N.a. | N.a. | CAARMS | N.a. | N.a. (16-30) | 2 | Period of care was extended if patients were not stable or had socio-occupational difficulties that it was considered could make an extension beneficial for them. |
| Tay et al, 2015^13^ | Support for Wellness Achievement Programme (SWAP) | Singapore | 161 | 80.6% APS, 2.9% BLIPS, 28% GRD | CAARMS | 69.7 | 21, 3.5 (16-30) | 5 | 20% had comorbid anxiety disorders, 34.8% depressive disorders 11.6% adjustment disorder and 7.1% bipolar disorder. 25.8% suffered no comorbidity. Impairment functioning was moderate. 14.8% CHR-P were given antipsychotic medications in doses of Haloperidol equivalent 0.5 to 1.5 mg per day. The most common antipsychotic prescribed was risperidone. |
| Coates et al, 2019^14^ | EIP in New South Wales | Australia | 19 | N.a. | N.a. | 64.2^b^ | 18 (12-24)^b^ | 3 | 24.2% had no family or carer involved in care, the most significant barrier was families not wanting to be involved (71.4%). Most common non-psychotic comorbidities were anxiety (60.6%), depression (42.4%), trauma (33.3%), attachment issues (30.3%) and developmental delays (24.2%); 69.7% reported using illicit drugs; 72.7% of patients lived with one or both parents and 60.1% had history of family mental illness; 48.7% were identified as at risk of metabolic syndrome. |
| Penno et al, 2017^15^ | Early Psychosis Program (EPP) service Hawthorn Melbourne | Australia | 4 | N.a. | N.a. | 60.7 | 31^b^, 11.9 (16-65)^b^ | 2 | FEP individuals and individuals treated for less than 18 months were included (16-64 years old). CHR-P individuals composed 1.67% of the individuals in the service. Behaviour, impairment, symptoms and social functioning improved after the intervention. Improvement in the substance use items in the behavioural subscale was significant in the first phase of treatment but did not continue with time. |
| Geros, 2020^16^ | Personal Assessment and Crisis Evaluation (PACE) | Australia | 467 | N.a. | CAARMS | 44.3 | 18.7 (15-24) | 4 | 18.6% CHR-P individuals transitioned to psychosis after median 253 days follow-up. |
| Nelson et al, 2013^17^ | Personal Assessment and Crisis Evaluation (PACE) | Australia | 416 | 79.4% APS, 14.1% BLIPS, 28.9% GRD | CAARMS | 48.1 | 18.9, 3.4 (15-30) | 5 | Highest rate of transition was within the first two years: 20.4%; It reached 34.9% after ten years. Baseline functioning and duration of symptoms prior to entry were predictive variables of conversion. For every year of symptoms prior to clinic entry the risk the likelihood of transition increased by 12%. Baseline disorder of thought content and baseline negative symptoms were significantly associated with transition. |
| Phillips et al, 2002^18^ | Personal Assessment and Crisis Evaluation (PACE) | Australia | 36 | N.a. | CAARMS | N.a. | N.a. (14-30) | 2 | The specific preventive intervention decreased the rate of psychosis at the end of the 6-month treatment phase. Overall psychopathology improved. |
| Yung et al, 2007^19^ | Personal Assessment and Crisis Evaluation (PACE) | Australia | 119 | N.a. | N.a. | N.a. | N.a. (14-25) | 1 | Rate of transition to psychosis within 12 months was 35%. The primary diagnostic outcome of the group who developed psychosis was schizophrenia (65%). |
| Yung et al, 1998^20^ | Personal Assessment and Crisis Evaluation (PACE) | Australia | 20 | N.a. | BPRS, SANS, CAARMS | N.a. | N.a. | 2 | 16.3 % of appropriate accepted referrals refused any follow-up. Transition rate was 40% after six months; 83.6% transitioned within the first month. |
| Yung et al, 1998^21^ | Personal Assessment and Crisis Evaluation (PACE) | Australia | 23 | 74% APS, 13.2% BLIPS, 39.2% GRD | BPRS | N.a. | N.a. (16-30) | 3 | Dropout at 12 months was 17.4%. Transition rate over 12 months was 48%, over 6 months was 40%. |
| Yung et al, 2006^22^ | Personal Assessment and Crisis Evaluation (PACE) | Australia | 119 | 93.3% APS, 10.9% GRD | CAARMS | 49 | 18.1 (15-24)^b^ | 5 | Six months rate was 10.1%; those who developed psychosis by six months had significantly lower functioning. |
| Yung et al, 2008^23^ | Personal Assessment and Crisis Evaluation (PACE) | Australia | 119 | 93.3% APS, 10.9% GRD | CAARMS | 49 | 18.1 (15-24)^b^ | 5 | A central triage service took referrals for all three service components and referred to a specific sub-program depending on clinical judgement. In the CHR-P group two years transition rate was 16%, six-months rate was 10.1%; CHR-P individuals had greater odds of transition (OR=16.2) than individuals n fulfilling CHR-P criteria. |
| Carr et al, 2000^24^ | Psychological Assistance  Service (PAS) | Australia | 60 | 48.3% APS, 36.7% BLIPS, 15% GRD | SANS, BPRS | 61.7 | 17.6 | 3 | 35% CHR-P suffered major depression, 6.7% dysthymia, 5% bipolar disorder, 5% social phobia and 6.7% OCD. 9% CHR-P individuals transitioned after 14.6 months. |
| Conrad et al, 2014^25^ | Psychological Assistance  Service (PAS) | Australia | 191 | 69.1% APS, 26.2% GRD, 16.2% BLIPS | CAARMS | 57.1 | 17.6, 3 (12-25) | 4 | Psychotic individuals had the highest aggregate contact rates per client per year (1.25), while the CHR-P and recent psychosis groups had the highest level of psychological assistance service contact during the presentation window (3.16 and 2.96 contacts, respectively). 62.3% CHR-P suffered depression, 42.9% anxiety, 20.9% personality disorders, 6.8% substance misuse, 67.5% other mental health problems and 6.3% physical disorders. 70.7% were under the age of 18 years at the service presentation. |
| Conrad et al, 2017^26^ | Psychological Assistance  Service (PAS) | Australia | 191 | 69.1% APS, 26.2% GRD, 16.2% BLIPS | CAARMS | 57.1 | 17.6, 3 (12-25) | 5 | 66% CHR-P individuals had at least one problem recorded: 17.3% psychosis, 19.9% depression, 16.2% anxiety, 15.2% substance misuse, 8.9% personality disorder, 19.3% physical problems. CHR-P individuals had a transition rate of 17.3% after two years. |
| Leuci et al, 2019^27^ | Parma- Early Psychosis” program | Italy | 138 | 91.3% APS, 4.3% BLIPS, 4.3% GRD | CAARMS | 58.9%^b^ | 36.3, 11.1^b^ (12-35)^c^ | 5 | Major depression (without psychotic symptoms) was the most frequent diagnosis at baseline in CHR-P individuals (56.6%), followed by bipolar disorder (34.8%), schizotypal personality disorder (4.3%) and brief psychotic disorder (4.3%). |
| Kotlicka-Antczak et al, 2018^28^ | Programme of Recognition and Therapy (PORT) | Poland | 99 | 76.2% APS, 4% BLIPS, 37.4% GRD | CAARMS | 45.4 | 19, 3.6 (15-32) | 4 | 19.2% CHR-P individuals dropped out during follow-up. Most common reasons for dropping out were stigmatization (52.6%), lack of acceptance of the proposed treatment (47.4%) and change of residence (31.6%). Half of the respondents reported their place of residence as the city in which the main information campaign of the service was carried out. 64% received a comorbid DSM-IV Axis I diagnosis. Most cost common diagnoses were depressive disorders (44.4%) and anxiety disorders (19%). 28.3% of the sample met the criteria for personality disorders, including schizotypal personality disorders (20.2% of participants), followed by mixed (5%) and borderline personality disorders (3%). |
| Kotlicka-Antczak et al, 2015^29^ | Programme of Recognition and Therapy (PORT) | Poland | 81 | N.a. | CAARMS | N.a. | N.a (15-29) | 2 | 19.75% dropped out of the service. The main reasons for doing so were change of residence including emigration (6.2%), lack of acceptance of the proposed treatment (11.1%) and stigmatization problems (12.3%). It is suggested that locating PORT in a hospital with psychiatric clinics could be the key cause of stigmatization  18.5% individuals converted into psychosis, based on the overall rate within the years 2010–2013. |
| Meneghelli et al, 2010^30^ | Programma 2000 Milan | Italy | 81 | N.a. | ERIraos, BPRS | 70.4 | 22.3, 3.6 (17-30) | 5 | After one year, information from 70.4% of patients was retrieved. After one year, 12.3% of CHR-P group were positive for the core symptoms of schizophrenia; 3.5%, received a formal diagnosis. Second-generation antipsychotic compounds were prescribed to 42% of CHR-P individuals. |
| Cocchi et al, 2013^31^ | Programma 2000 Milan | Italy | 96 | N.a. | ERIraos, BPRS | 68 | 22.1, 3.6 | 4 | The establishment of an EIP in a large metropolitan area such as Milan led to an increase of referrals from people and agencies that were not directly involved in the mental health care system, also attracting patients with longer DUI and DUP than those who habitually access psychiatric services. Duration of untreated illness was 30.7 months in CHR-P individuals. 9% CHR-P suffered a history of attempted suicide. 60% CHR-P had a family psychiatric history (21% psychosis, 43% affective disorder, 14% substance abuse, 12% personality disorder. |
| Katsura et al, 2014^32^ | Sendai ARMS  and first episode (SAFE) clinic | Japan | 106 | 95.3% APS, 3.8% BLIPS, 14.1% GRD | CAARMS | 37.7 | 20, 4.3 (14-35) | 4 | 25.4% discontinued treatment at the SAFE clinic before 12 months. 85% CHR-P had been seen by at least one psychiatrist. 13.2% transitioned to psychosis (10 within the first year, 3 in the following year, and 1 after 836 days). 37.3% took AP before intake and 26.5% of them (71% of the previously treated) continuously received AP. Of the AP-naive, 32.7% began receiving them at the SAFE clinic. |
| Tiffin et al, 2007^33^ | Teesside EIP Service | United Kingdom | 9 | N.a. | CAARMS | N.a. | (14-18) | 2 | 33.3% CHR-P individuals converted to psychosis (unclear period). |
| Pelizza et al, 2019^34^ | Reggio Emilia At-Risk Mental States (ReARMS) | Italy | 79 | 91.1% APS, 3.8% BLIPS, 5.1% GRD | CAARMS | 45.6 | 18.6, 6.4 (13-35) | 5 | Out of the whole sample (CHR-P+FEP individuals), 9.3% refused treatment and less individuals dropped out during the first year of treatment (15.6%) compared to pre-implementation of the service protocol (35.2%) (p=001).  In CHR-P individuals, major depression (without psychotic features) was the most frequent diagnosis (50.6%) at initial examination, followed by anxiety disorders (27.8%), schizotypal personality disorder (17.7%) and brief psychotic disorder (3.9%). |
| Pelizza et al, 2020^35^ | Reggio Emilia At-Risk Mental States (ReARMS) | Italy | 70 | N.a. | CAARMS | 45.7 | 18.5, 4.5 (13-35) | 4 | 14.5% CHR-P had a a history of previous attempted suicide. 16.7% suffered a suicide attempt in the next two years. CHR-P individuals had more severe suicidal ideation than FEP and non CHR-P. 11.4% of the total sample transitioned to psychosis after two years. |
| Pelizza et al, 2020^36^ | Reggio Emilia At-Risk Mental States (ReARMS) | Italy | 51 | N.a. | CAARMS | 39.2 | 15.6 (1.6) (13-35) | 3 | Social cognition deficits are prominent in CHR-P adolescents. 23.5% CHR-P after one year and 52.9% after two years did not complete the follow-up. |
| Pelizza et al, 2019^37^ | Reggio Emilia At-Risk Mental States (ReARMS) | Italy | 44 | N.a. | CAARMS | 40.9 | 15.4, 1.6 (13-35) | 4 | 9.3% CHR-P refused, and 15.6% dropped out during the first year of treatment. Individuals were mainly referred by emergency room/general hospitals (24%), GPs (33.3%), or were self-referred (15%) |
| Pelizza et al, 2019^38^ | Reggio Emilia At-Risk Mental States (ReARMS) | Italy | 79 | N.a. | CAARMS | 45.6 | 18.6 (4.4) (13-35) | 4 | Compared to those not fulfilling CHR-P criteria, CHR-P individuals had higher introvertive anhedonia scores. 15.2% CHR-P abandoned the study before one year. |
| Adamson et al, 2018^39^ | Lincolnshire Early Intervention in Psychosis | United Kingdom | 138 | 85.5% APS, 12.3% BLIPS, 2.2% GRD | CAARMS | 65 | 27.4, 11 (14-65)^b^ | 3 | The service received 406 referrals. The mean average length of time from referral to treatment was 1.5 weeks, with a mean average of 88% of people being seen within 2 weeks from the date of referral; a total of 261 individuals were finally accepted on the team caseload. Most referrals came from the GP (31%). 22% individuals were over 35 years old. |
| McFarlane et al, 2010^40^ | Portland  Identification and Early Referral (PIER) | United States of America | 148 | N.a. | SIPS/SOPS | 53 | 16.5, 3.1 (12-35) | 5 | 7% CHR-P individuals declined treatment, 80% were treated ≥3 months. 13% dropped out in <3 months. Community and professional education had a lagged effect on referrals of about six months. |
| Power et al, 2007^41^ | Outreach & Support in South London (OASIS) | United Kingdom | 292 | N.a. | N.a. | 53.8 | 24.1, 4.5 (16-35) | 5 | The engagement with the service was good, and 85% of clients remained well engaged with the service. Their mean DUP was 11 days. |
| Valmaggia et al, 2009^42^ | Outreach & Support in South London (OASIS) | United Kingdom | 114 | N.a. | CAARMS | 58.8 | 24, 4.7 | 5 | 13.1% refused any intervention after the initial assessment. 21% CHR-P individuals developed a FEP. The mean DUP in those who made a transition was 10.8 days. Most clients who made a transition did not need admission (63%), 8% were sectioned and 29% were admitted informally within 1 month of transition. After 24 months the costs of OASIS service was lower than care as usual. |
| Fusar-Poli et al, 2015^43^ | Outreach & Support in South London (OASIS) | United Kingdom | 258 | 70.2% APS, 8.5% BLIP, 21.3% combined | CAARMS | 56.6 | 22.9, 4.5 (14-53) | 5 | CBT was associated with good outcomes when used as first-line therapy, 62% completed 12 sessions. At follow-up, the majority 63% of the patients were medication-free, and 60% of patients had a good functional status. The average time of follow-up was 6 years. 18% made a transition to psychosis: 12.8% developed a bipolar psychotic disorder; 61.7% a schizophrenia spectrum disorder; and 25.5% developed a psychotic disorder not otherwise specified. |
| Green et al, 2011^44^ | Outreach & Support in South London (OASIS) | United Kingdom | 137 | N.a. | CAARMS | 57.3 | 23.7, 4.7 (15-35) | 5 | 17.4% declined an assessment. Of those that were accepted, 88% engaged with the service. Those that engaged were more frequently employed compared to non-attenders and individuals that disengaged. There were no differences between the engaged group, nonattenders and those that disengaged CHR-P groups in terms of age, ethnicity, place of birth, marital status or referral source. Of those that disengaged, 22.6% received of a psychotic diagnosis 10 months after referred to the service and 69.4% presented to other mental health services. |
| Broome et al, 2005^45^ | Outreach & Support in South London (OASIS) | United Kingdom | 58 | 84.4% APS, 20.7% BLIP, 13.7% GRD | CAARMS | 65.5 | 24.1, 4.2 (14-35) | 4 | Clients and their relatives were generally happy with the service offered, particularly with the clinical contact being outside traditional mental health settings and with the staff were being flexible about the timing of appointments. Primary care clinicians liked having clients seen in their surgery and liked the accessibility of the service. 10.3% individuals developed psychosis. Mean delay in treatment after transition was 12 days (range 7-21 days). 66.7% of those required admission and in 16.7% an assessment for compulsory admission under the UK Mental Health Act was needed. 56.8% had additional psychiatric morbidity in addition to their CHR-P symptoms. |
| Fusar-Poli et al, 2013^46^ | Outreach & Support in South London (OASIS) | United Kingdom | 290 | 89% APS, 18% BLIPS, 14% GRD | CAARMS | 56.1 | 22.9, 4.6 (14-35) | 5 | 32% of the individuals assessed by the service were already psychotic at the time of the initial assessment. They were immediately referred to the FEP service. OASIS team produced significant savings mainly associated with the prevention of transition to psychosis and the benefits associated with a short duration of untreated psychosis. Over the 2-year follow-up period, 15.2% transitioned; mean time to transition was 375 days. Common comorbidities in CHR-P individuals were 21% depression, 10% substance use, 8% anxiety or social anxiety, 6% depression and anxiety, 5% PD and 3% OCD. |
| Tognin, 2019^47^ | Outreach & Support in South London (OASIS) | United Kingdom | 70 | N.a. | CAARMS | 57 | 22.9, 5.5 (14-36) | 4 | 80% CHR-P individuals had access to vocational support. In 85%, when vocational support was offered, it was offered early on, within 6 months of acceptance to the services. 23.3% of clients were in mainstream education and 28% were working part or full-time. |
| Kwon, 2012^48^ | Seoul Youth Clinic | Korea | 92 | 91.3% APS, 1% BLIPS, 18.3% GRD | CAARMS, SIPS, PANSS | 67.3 | 20.6, 0.4 (15-35) | 4 | After 39 months 16.3% converted to psychosis. 64.1% have comorbid mood disorder, 46.7% anxiety disorder, and 21.7% personality disorders. |
| Gaspar et al, 2018^49^ | University of Chile High-risk Intervention Program (UCHIP) | Chile | 27 | 92.6% APS, 7.4% GRD | SIPS/SOPS | 70.3 | 17.6 (12-35) | 3 | 100% CHR-P individuals received psychoeducation and 62.9% received CBT. 85.2% CHR-P individuals were prescribed daily, low-dose second generation antipsychotics. 5% were prescribed anxiolytics, 12% antidepressant medication and 22% omega-3 polyunsaturated fatty acids. 14.8% CHR-P individuals suffered anxiety disorders and 44.4% mood disorders. 22% of the CHR-P individuals met criteria for threshold psychosis and 18% for personality disorder within two years. Mean time to transition was 348.9 days and median time of transition was 331 days. |

^a^Not mutually exclusive; ^b^ Overall sample, not only included CHR-P; ^c^ CHR-P individuals.

APS: Attenuated psychosis syndrome; BLIPS: Brief Limited Intermittent Psychotic Symptoms; BPRS: Brief Psychiatric Rating Scale; CAARMS: Comprehensive Assessment for at Risk Mental States; CASH: comprehensive Assessment of Symptoms and History; ERIraos: Early Recognition Inventory Retrospective Assessment of Symptoms; GRD: Genetic Risk and Deterioration Syndrome HAM-A: Hamilton Anxiety Rating Scale ; HOPES: Hunter Opinions and Personal Expectations Scale; PANSS: Positive and Negative Syndrome Scale; RSM: Rating Scale for Mania; SAPS: Scale for the Assessment of Positive Symptoms; SANS: Scale for Assessment of Negative Symptoms; SOFAS: Social and Occupational Functioning Assessment Scale.

**eTable 3: Main characteristics of CHR-P individuals in multi-site studies**

| **Author, year** | **Name of the**  **CHR-P service** | **Country** | **CHR-P users description** | | | | | **Key findings** |
| --- | --- | --- | --- | --- | --- | --- | --- | --- |
|  |  |  | **Sample size** | **Subgrups** | **CHR-P assessment tool** | **Sex (% male** | **Age: mean, SD (range)** |  |
| Lynch et al, 2016^50^ | Early Detection, Intervention and Prevention of Psychosis Program (EDIPPP)^b^ | United States | 205 | N.a | SIPS/SOPS | 60^a^ | 16.4, 3.3 | School, mental health, and medical practitioners were the critical audiences for community outreach. Consistency of outreach effort resulted in more referrals. Gaining access to primary care providers was a challenge because of scepticism about prevalence of psychotic illnesses and limited time available to focus on mental health issues. All sites yielded appropriate referrals of at-risk youths. |
| McFarlane et al, 2012^51^ | Early Detection, Intervention and Prevention of Psychosis Program (EDIPPP)^b^ | United States | 205 | N.a | SIPS/SOPS | 57 | 16.4, 3.3 (12-25) | Frequent comorbidities for CHR-P individuals were found: 86% AXIS-I disorder, 50% mood disorder and 42% anxiety disorder. |
| Cocchi et al, 2015^52^ | Early detection and intervention in psychosis. A preventive approach to schizophrenia^c^ | Italy | 24 | N.a | ERIraos-CL | 79 | 21.2, 4.7 (17-30) | Family history of psychopathology was reported in 57% of CHR-P patients. There was an improvement in psychotic psychopathology in CHR-P individuals at 1-year follow up. No dropouts from the program after one year. |
| Fusar-Poli et al, 2019^53^ | Pan-London Network for Psychosis Prevention (PNP), OASIS Lambeth & Southwark | United Kingdom | 419 | 80% APS, 19.59% BLIPS/BIPS, 0.41% GRD | CAARMS | 54.6 | 22.8, 4.9 (14-35) | Transition risk across the PNP (n=787, from OASIS, THEDS, NEIS, HEADS UP, and 38 users from Bedfordshire and Lutton) was 14.1% after one year, 22.1% after two years, 28.1% after three years, and 30.1% after four years. |
|  | Pan-London Network for Psychosis Prevention (PNP), OASIS Croydon & Lewisham | United Kingdom | 159 | 75.72% APS, 19.65% BLIPS/BIPS, 4.62% GRD | CAARMS | 57.1 | 22, 4.8 (14-35) | N.a. |
|  | Pan-London Network for Psychosis Prevention (PNP), Tower Hamlets Early Detection Service (THEDS) | United Kingdom | 104 | Most in the APS subgroup. | SIPS/SOPS | 69.2 | 22, 4 (16-25) | Self-reported substance misuse present in 40% of service users (cannabis the most common). Mood disorders are the most prevalent diagnosed comorbid condition (56% of total comorbidities), followed by substance misuse (10%), and anxiety disorders (7%). |
|  | Pan-London Network for Psychosis Prevention (PNP), City & Hackney At-Risk Mental State Service (HEADS UP) | United Kingdom | 31 | 51.61% APS, 32.26% BLIPS/BIPS, 16.13% GRD | CAARMS | 58.1 | 23.3, 4.9 (18-35) | Substance misuse present in 45% of service users (cannabis being the most common). Most service users (77%) have received CBT-informed individual psychotherapy, and less frequently (19%), family therapy. About half of service users receive psychopharmacological treatment (antidepressants most frequently). 3.2% service users received low-dose antipsychotic medication. |
|  | Pan-London Network for Psychosis Prevention (PNP), Newham Early Intervention Service (NEIS) site | United Kingdom | 36 | 55.56% APS, 25% BLIPS/BIPS, 19.44% GRD | CAARMS | 69.4 | 22.3, 3.7 (18-35) | Better engagement reported for CHR-P individuals compared to FEP patients. 27.8% presented with substance use. |
| Simon et al, 2012^54^ | The Swiss Early Psychosis Project (SWEPP), FePsy | Switzerland | N.a | N.a | N.a | N.a | Adolescents and adults | A multi-modal education approach and a solid communication network were successful in increasing EP services coverage in Switzerland. |
|  | SWEPP, Bruderholz Early Psychosis  Outpatient Service | Switzerland | N.a | N.a | N.a | N.a | Adolescents and adults | N.a |
|  | SWEPP, ZInEP | Switzerland | N.a | N.a | N.a | N.a | 13-35 | N.a |
|  | SWEPP, FETZ | Switzerland | N.a | N.a | N.a | N.a | Children, adolescents  and adults | N.a |
|  | SWEPP, Early Psychosis Outpatient Service, Aargau | Switzerland | N.a | N.a | N.a | N.a | Adolescents  and adults | N.a |
|  | SWEPP, Station FP, Münsterlingen | Switzerland | N.a | N.a | N.a | N.a | 16–25 | N.a |
|  | SWEPP, FES | Switzerland | N.a | N.a | N.a | N.a | 16–35 | N.a |
|  | SWEPP, Treatment and early intervention  in Psychosis Program, Lausanne | Switzerland | N.a | N.a | N.a | N.a | 18–35 | N.a |
|  | SWEPP, JADE | Switzerland | N.a | N.a | N.a | N.a | 18–25 | N.a |
| Bertulies-Esposito 2020^55^ | Service #3 | Canada | N.a | N.a | N.a | N.a | 14-35 | Most services accepted self-referrals and referrals from schools, family, and friends. Time between referral and intake was from 3 to 90 days. Most programs offered pharmacotherapy, patient and family psychoeducation, cognitive-behavior therapy, and substance misuse interventions. |
|  | Service #9 | Canada | N.a | N.a | N.a | N.a | 14-28 |  |
|  | Service #12 | Canada | N.a | N.a | N.a | N.a | 18-35 |  |
|  | Service #13 | Canada | N.a | N.a | N.a | N.a | 17-35 |  |
|  | Service #16 | Canada | N.a | N.a | N.a | N.a | <17 |  |
|  | Service #17 | Canada | N.a | N.a | N.a | N.a | 6-17 |  |
| Ruff 2012^56^ | PIER | United States | N.a | N.a | N.a | N.a | N.a. | 37% community referrals were at CHR-P. Community educational presentations were associated with referrals six months later. |

APS: Attenuated psychosis syndrome; BLIPS: Brief Limited Intermittent Psychotic Symptoms; BPRS: Brief Psychiatric Rating Scale; CAARMS: Comprehensive Assessment for at Risk Mental States; CHR-P: clinical high risk of psychosis; ERIraos: Early Recognition Inventory Retrospective Assessment of Symptoms; FEP: first episode of psychosis; GRD: Genetic Risk and Deterioration Syndrome; PANSS: Positive and Negative Syndrome Scale; SANS: Scale for Assessment of Negative Symptoms; SIPS: Structured Interview for Prodromal Symptoms; SOPS: Scale of Prodromal Symptoms.

^a^ Overall EI sample (CHR-P + FEP), not only included CHR-P; ^b^ Portland Identification and Early Referral (PIER), Early Assessment and Support Team (EAST), Michigan Prevents Prodromal Progression (M3P), Recognition and Prevention (RAP), Early Detection and Preventive Treatment (EDAPT), Early Assessment and Resource Linkage for Youth (EARLY); ^c^ Milan (Programma 2000), Rome (area D), Grosseto, Salerno (area 1, Nocera), Catanzaro (Operative Unit of Soverato).

**eMethods 1: Questions** **Mixed Methods Appraisal Tool (MMAT)**

1. Are the participants representative of the target population?

2. Was recruitment period long enough?

3. Are measurements appropriate regarding both the outcome and intervention (or exposure)?

4. Were individuals in the study comprehensively characterized?

5. Were individuals followed long enough for outcomes to occur and improvements to be noticed?

**eResults 1:** **Details about service configuration, outreach strategy and referrals**

**Professionals involved:**

^a^ Psychiatrist: may include scientific consultant, neuropsychiatrist, adult psychiatrist, child and adolescent psychiatrist, resident or training psychiatrists.

^b^ Clinical psychologist or counsellor: may include clinical psychologists, psychotherapist, assistant psychologist, training psychologist, psychologist NOS or clinical counsellor.

^c^ Case manager/care coordinator: may include clinical coordinator, case manager, or team leader.

^d^ Nurse: may include psychiatric nurse or CAMH trained nurse.

^e^ Research personnel: may include honorary research associates, research assistants or PhD students.

**Outreach activities:**

^f^ Targeting education professionals: may include school and university counsellors, psychopedagogy attention services, primary and secondary education centres or college and university healthcare services.

^g^ Service promotion to NGOs and community services: may include community health centres, non-government youth centres, mental health charities and volunteer organisations, local pastoral services, youth services or multi-cultural groups.

^h^ Service promotion to social and governmental services: may include preventive attention teams, primary social care attention, justice institutions for the protection of youth in risk situation or law enforcement.

^i^ Print and other media: may include information brochures, posters, articles in professional journals and newsletter, local newspapers, scientific conferences, leaflets and newsletters or promotional videos.

**Referral sources**

^j^ Outpatient or community mental health services: may include non-acute mental health services, drug and alcohol services, adult community mental health services, child and youth mental health services, psychosocial counselling services, local outpatient unit, eating disorder care service, primary care psychology, external or private mental health professional (psychologists, psychiatrists), or mental health sources NOS.

^k^ General healthcare: may include GPs, community health centre or general hospital, private doctors or policlinic doctors.

^l^ Education organisations or services^:^ may include school counselling services, psycho-pedagogy attention team, school nurse, university health centres or learning disability services.

^m^ Inpatient mental health services: may include psychiatric hospital or department of short hospitalization.

^n^ Government organisations: may include police and criminal system, youth justice system, Armed Forces or prison services.

^o^ Community organisations: may include NGOs, youth services, family services, department of community services, third sector organisations or community forensic services.

**eResults 2:** **Details about interventions and outcomes**

**Interventions**

^a^ Case management and psychosocial support: may include supported employment and education, recreation, housing, medication management, needs-based support, occupational therapy, school coordination.

^b^ Family interventions: may include multifamily group, family support, brief family interventions, family therapy, family counselling, behavioural family therapy.

^c^ CBT-based intervention: may include cognitive-behavioural therapy for psychosis (CBTp) or stress-reduction CBT, social CBT or ACT.

^d^ Oher individual psychotherapeutic interventions: may include individual motivational sessions, supportive counselling, motivational interviewing, relaxation training, cognitive remediation, solution focused brief therapy, social skills training, substance misuse work, psychotherapy NOS.

^e^ Physical health interventions: may include nutritional help, medical reviews, physical health interventions NOS.

^f^ Group social or therapeutic interventions: may include therapeutic or social group activities, including music, multimedia and computer training.

**Outcomes**

^g^ Administrative data: including hospital admissions and compulsory admissions.

**REFERENCES**

1. Oppetit A *et al.* The C'JAAD: a French team for early intervention in psychosis in Paris. *Early Intervention in Psychiatry* 2018; **12**(2)**:** 243-249.

2. Spada G *et al.* Identifying children and adolescents at ultra high risk of psychosis in Italian neuropsychiatry services: a feasibility study. *European Child & Adolescent Psychiatry* 2016; **25**(1)**:** 91-106.

3. Pruessner M *et al.* The Clinic for Assessment of Youth at Risk (CAYR): 10 years of service delivery and research targeting the prevention of psychosis in Montreal, Canada. *Early Intervention in Psychiatry* 2017; **11**(2)**:** 177-184.

4. Quijada Y, Tizon JL, Artigue J, Parra B. At-risk mental state (ARMS) detection in a community service center for early attention to psychosis in Barcelona. *Early Intervention in Psychiatry* 2010; **4**(3)**:** 257-262.

5. Vallina-Fernández O *et al.* Aplicación de un programa de intervención temprana en psicosis: un nuevo desarrollo para las unidades de salud mental. *Avances en salud mental relacional* 2003; **2**.

6. Kollias C *et al.* Early psychosis intervention outpatient service of the 1st Psychiatric University Clinic in Athens: 3 Years of experience. *Early Intervention in Psychiatry* 2018; **12**(3)**:** 491-496.

7. Leanza L *et al.* Predictors of study drop-out and service disengagement in patients at clinical high risk for psychosis. *Soc Psychiatry Psychiatr Epidemiol* 2020; **55**(5)**:** 539-548.

8. Riecher-Rossler A *et al.* The Basel early-detection-of-psychosis (FEPSY)-study - design and preliminary results. *Acta Psychiatrica Scandinavica* 2007; **115**(2)**:** 114-125.

9. Schultze-Lutter F, Hubl D, Schimmelmann BG, Michel C. Age effect on prevalence of ultra-high risk for psychosis symptoms: replication in a clinical sample of an early detection of psychosis service. *European Child & Adolescent Psychiatry* 2017; **26**(11)**:** 1401-1405.

10. Theodoridou A *et al.* Early Recognition of High Risk of Bipolar Disorder and Psychosis: An Overview of the ZInEP "Early Recognition" Study. *Front Public Health* 2014; **2:** 166.

11. Schultze-Lutter F, Ruhrmann S, Klosterkotter J. Early detection of psychosis - Establishing a service for persons at risk. *European Psychiatry* 2009; **24**(1)**:** 1-10.

12. Rao S *et al.* Support for Wellness Achievement Programme (SWAP): A Service for Individuals with At-Risk Mental State in Singapore. *Annals Academy of Medicine Singapore* 2013; **42**(10)**:** 552-555.

13. Tay SA *et al.* Support for Wellness Achievement Programme (SWAP): clinical and demographic characteristics of young people with at-risk mental state in Singapore. *Early Intervention in Psychiatry* 2015; **9**(6)**:** 516-522.

14. Coates D, Wright L, Moore T, Pinnell S, Merillo C. The psychiatric, psychosocial and physical health profile of young people with early psychosis: Data from an early psychosis intervention service. *Child & Youth Services* 2019; **40**(1)**:** 93-115.

15. Penno SJ, Hamilton B, Petrakis M. Early Intervention in Psychosis: Health of the Nation Outcome Scales (HoNOS) Outcomes From a Five-Year Prospective Study. *Archives of Psychiatric Nursing* 2017; **31**(6)**:** 553-560.

16. Geros H *et al.* Migrant status and identification as ultra-high risk for psychosis and transitioning to a psychotic disorder. *Acta Psychiatr Scand* 2020; **141**(1)**:** 52-59.

17. Nelson B *et al.* Long-term Follow-up of a Group at Ultra High Risk ("Prodromal") for Psychosis The PACE 400 Study. *Jama Psychiatry* 2013; **70**(8)**:** 793-802.

18. Phillips L *et al.* The PACE Clinic: Identification and Management of Young People at “Ultra” High Risk of Psychosis. *J Psychiatr Pract* 2002; **8**(5)**:** 255-269.

19. Yung AR *et al.* PACE: a specialised service for young people at risk of psychotic disorders. *Medical Journal of Australia* 2007; **187**(7)**:** S43-S46.

20. Yung AR *et al.* Prediction of psychosis - A step towards indicated prevention of schizophrenia. *British Journal of Psychiatry* 1998; **172:** 14-20.

21. Yung AR *et al.* Can we predict the onset of first-episode psychosis in a high-risk group? *International Clinical Psychopharmacology* 1998; **13:** S23-S30.

22. Yung AR *et al.* Testing the Ultra High Risk (prodromal) criteria for the prediction of psychosis in a clinical sample of young people. *Schizophrenia Research* 2006; **84**(1)**:** 57-66.

23. Yung AR *et al.* Validation of "prodromal" criteria to detect individuals at ultra high risk of psychosis: 2 year follow-up. *Schizophrenia Research* 2008; **105**(1-3)**:** 10-17.

24. Carr V *et al*. A risk factor screening and assessment protocol for schizophrenia and related psychosis. *Australian and New Zealand Journal of Psychiatry* 2000; **34:** S170-S180.

25. Conrad AM *et al.* Ten-year audit of clients presenting to a specialised service for young people experiencing or at increased risk for psychosis. *Bmc Psychiatry* 2014; **14**.

26. Conrad AM *et al.* Utility of risk-status for predicting psychosis and related outcomes: evaluation of a 10-year cohort of presenters to a specialised early psychosis community mental health service. *Psychiatry Research* 2017; **247:** 336-344.

27. Leuci E, Quattrone E, Pellegrini P, Pelizza L. The "Parma-Early Psychosis" program: General description and process analysis after 5 years of clinical activity. *Early Interv Psychiatry* 2019.

28. Kotlicka-Antczak M *et al* Polish individuals with an at-risk mental state: demographic and clinical characteristics. *Early Intervention in Psychiatry* 2018; **12**(3)**:** 391-399.

29. Kotlicka-Antczak M, Pawelczyk T, Rabe-Jablonska J, Pawelczyk A. PORT (Programme of Recognition and Therapy): the first Polish recognition and treatment programme for patients with an at-risk mental state. *Early Intervention in Psychiatry* 2015; **9**(4)**:** 339-342.

30. Meneghelli A, Cocchi A, Preti A. 'Programma2000': a multi-modal pilot programme on early intervention in psychosis underway in Italy since 1999. *Early Intervention in Psychiatry* 2010; **4**(1)**:** 97-103.

31. Cocchi A *et al* Patterns of referral in first-episode schizophrenia and ultra high-risk individuals: results from an early intervention program in Italy. *Social Psychiatry and Psychiatric Epidemiology* 2013; **48**(12)**:** 1905-1916.

32. Katsura M *et al.* A naturalistic longitudinal study of at-risk mental state with a 2.4 year follow-up at a specialized clinic setting in Japan. *Schizophrenia Research* 2014; **158**(1-3)**:** 32-38.

33. Tiffin PA, Hudson S. An Early Intervention in Psychosis Service for adolescents. *Early Intervention in Psychiatry* 2007; **1**(2)**:** 212-218.

34. Pelizza L *et al.* The "Reggio Emilia At-Risk Mental States" program: A diffused, "liquid" model of early intervention in psychosis implemented in an Italian Department of Mental Health. *Early Interv Psychiatry* 2019; **13**(6)**:** 1513-1524.

35. Pelizza L *et al.* Suicide risk in young people at Ultra-High Risk (UHR) of psychosis: Findings from a 2-year longitudinal study. *Schizophr Res* 2020.

36. Pelizza L *et al.* Subjective experience of social cognition in adolescents at ultra-high risk of psychosis: findings from a 24-month follow-up study. *Eur Child Adolesc Psychiatry* 2020.

37. Pelizza L *et al.* Anhedonia in adolescents at ultra-high risk (UHR) of psychosis: findings from a 1-year longitudinal study. *Eur Arch Psychiatry Clin Neurosci* 2020; **270**(3)**:** 337-350.

38. Pelizza L *et al.* Characterization of young people with first episode psychosis or at ultra-high risk: the Reggio Emilia At-Risk Mental States (ReARMS) program. *Riv Psichiatr* 2019; **54**(6)**:** 254-263.

39. Adamson V *et al.* Implementing the access and waiting time standard for early intervention in psychosis in the United Kingdom: An evaluation of referrals and post-assessment outcomes over the first year of operation. *Early Intervention in Psychiatry* 2018; **12**(5)**:** 979-986.

40. McFarlane WR *et al* Portland Identification and Early Referral: A Community-Based System for Identifying and Treating Youths at High Risk of Psychosis. *Psychiatric Services* 2010; **61**(5)**:** 512-515.

41. Power P *et al.* Lambeth Early Onset (LEO) and Outreach & Support in South London (OASIS) service. *Early Intervention in Psychiatry* 2007; **1**(1)**:** 97-103.

42. Valmaggia LR *et al.* Economic impact of early intervention in people at high risk of psychosis. *Psychological Medicine* 2009; **39**(10)**:** 1617-1626.

43. Fusar-Poli P *et al.* Antidepressant, antipsychotic and psychological interventions in subjects at high clinical risk for psychosis: OASIS 6-year naturalistic study. *Psychological Medicine* 2015; **45**(6)**:** 1327-1339.

44. Green CEL, McGuire PK, Ashworth M, Valmaggia LR. Outreach and Support in South London (OASIS). Outcomes of non-attenders to a service for people at high risk of psychosis: the case for a more assertive approach to assessment. *Psychological Medicine* 2011; **41**(2)**:** 243-250.

45. Broome MR *et al.* Outreach and support in south London (OASIS): implementation of a clinical service for prodromal psychosis and the at risk mental state. *European Psychiatry* 2005; **20**(5-6)**:** 372-378.

46. Fusar-Poli P, Byrne M, Badger S, Valmaggia LR, McGuire PK. Outreach and support in South London (OASIS), 2001-2011: Ten years of early diagnosis and treatment for young individuals at high clinical risk for psychosis. *European Psychiatry* 2013; **28**(5)**:** 315-326.

47. Tognin S *et al.* The Provision of Education and Employment Support At the Outreach and Support in South London (OASIS) Service for People at Clinical High Risk for Psychosis. *Front Psychiatry* 2019; **10:** 799.

48. Kwon JS, Byun MS, Lee TY, An SK. Early intervention in psychosis: Insights from Korea. *Asian J Psychiatr* 2012; **5**(1)**:** 98-105.

49. Gaspar PA *et al.* Early psychosis detection program in Chile: A first step for the South American challenge in psychosis research. *Early Intervention in Psychiatry* 2019; **13**(2)**:** 328-334.

50. Lynch S *et al.* Early Detection, Intervention and Prevention of Psychosis Program: Community Outreach and Early Identification at Six US Sites. *Psychiatric Services* 2016; **67**(5)**:** 510-516.

51. McFarlane W, Cook W, Downing D, Ruff A, Lynch S *et al.* Early Detection, Intervention, and Prevention of Psychosis Program: Rationale, Design, and Sample Description. *Adolescent Psychiatry* 2012; **2**(2)**:** 112 - 124.

52. Cocchi A *et al.* Early intervention in psychosis: a feasibility study financed by the Italian Center on Control of Maladies. *Early Intervention in Psychiatry* 2015; **9**(2)**:** 163-171.

53. Fusar-Poli P *et al.* Pan-London Network for Psychosis-Prevention (PNP). *Front Psychiatry* 2019; **10:** 707.

54. Simon AE, Theodoridou A, Schimmelmann B, Schneider R, Conus P. The Swiss Early Psychosis Project SWEPP: a national network. *Early Intervention in Psychiatry* 2012; **6**(1)**:** 106-111.

55. Bertulies-Esposito B *et al.* Où en sommes-nous? An Overview of Successes and Challenges after 30 Years of Early Intervention Services for Psychosis in Quebec. *Can J Psychiatry* 2020**:** 706743719895193.

56. Ruff A, McFarlane W, Downing D, Cook W, Woodberry K. A Community Outreach and Education Model for Early Identification of Mental Illness in Young People. *Adolescent Psychiatry* 2012; **2:** 140-145.
